# Expression of colony-stimulating factor 1 is associated with occurrence of osteochondral change in pigmented villonodular synovitis

**DOI:** 10.1007/s13277-015-3197-5

**Published:** 2015-02-18

**Authors:** Takehiro Ota, Hiroshi Urakawa, Eiji Kozawa, Kunihiro Ikuta, Shunsuke Hamada, Satoshi Tsukushi, Yoshie Shimoyama, Naoki Ishiguro, Yoshihiro Nishida

**Affiliations:** 1 Department of Orthopaedic Surgery, Nagoya University Graduate School and School of Medicine, 65 Tsurumai, Showa, Nagoya, 466-8550 Japan; 2 Department of Pathology and Clinical Laboratories, Nagoya University Graduate School and School of Medicine, 65 Tsurumai, Showa, Nagoya, 466-8550 Japan

**Keywords:** Pigmented villonodular synovitis, CSF1, CSF1R, RANKL, Osteochondral change, Local recurrence

## Abstract

Pigmented villonodular synovitis (PVNS) is a benign, translocation-derived neoplasm. Because of its high local recurrence rate after surgery and occurrence of osteochondral destruction, a novel therapeutic target is required. The present study aimed to evaluate the significance of protein expression possibly associated with the pathogenesis during the clinical course of PVNS. In 40 cases of PVNS, positivity of colony-stimulated factor 1 (CSF1), its receptor (CSF1R), and receptor activator of nuclear factor kappa-B ligand (RANKL) were immunohistochemically determined. The relationship between the positivity and clinical outcomes was investigated. High positivity of CSF1 staining intensity was associated with an increased incidence of osteochondral lesions (bone erosion and osteoarthritis) (*p* = 0.009), but not with the rate of local recurrence. Positivity of CSF1R and RANKL staining was not associated with any clinical variables. The number of giant cells was not correlated with positivity of any of the three proteins, or with the clinical outcome. Focusing on knee cases, CSF1 positivity was also associated with the incidence of osteochondal change (*p* = 0.02). CSF1R positivity was high in cases which had local recurrence, but not significantly so (*p* = 0.129). Determination of CSF1 and CSF1R expression may be useful as a prognosticator of the clinical course and/or outcomes of PVNS.

## Introduction

Pigmented villonodular synovitis (PVNS), also known as diffuse-type giant cell tumor, is a rare proliferative synovial disease [1]. It usually occurs around the large joints such as the knees and hips, and often results in hemarthrosis and/or osteochondral destruction [2, 3]. Surgical resection is usually performed, but the recurrence rate is high [4]. Histologically, these tumors exhibit an infiltrative growth pattern and are composed of various proportions of mononuclear cells, multinuclear osteoclast-like giant cells, foamy macrophages, and siderophages [5]. The pathogenesis of PVNS, neoplastic or reactive proliferation, has been discussed. Vogrinic et al. concluded that giant cell tumor of tendon sheath is a non-neoplastic, polyclonal proliferative lesion [6]. On the other hand, cytogenetic analyses have revealed structural aberration of 1p11-13 in PVNS [7–9]. West et al. identified the translocation of chromosome 1 that involves the CSF1 gene with COL6A3 on chromosome 2. Additionally, FISH and immunohistochemical analyses indicated that a minority (2–16 %) of the cells harbor the translocation and overexpress CSF1. Meanwhile, overexpression of CSF1 receptor (CSF1R) positive cells has been observed in tumorous tissues [10] and high expression levels of CSF1 mRNA in PVNS [11]. These observations suggest that translocation of CSF1 and COL6A3 induces high expression of CSF1, which in turn attracts non-neoplastic cells which express CSF1R.

The colony-stimulating factor 1 (CSF1) is known as a cytokine that influences monocyte/macrophage differentiation and the inflammatory process [12, 13]. Myeloid lineage cells comprising monocytes, macrophages, and osteoclasts have the CSF1 receptor (CSF1R), and CSF1/CSF1R signaling pathway is essential to the growth and differentiation of these cells.

The multinucleated giant cells are variously expressed in the PVNS tissue. Taylor et al. showed that multinucleated giant cells have an osteoclast-like phenotype and are associated with bone resorption [14], which is one of the crucial events in PVNS. The multinucleated giant cells are formed by fusion of monocyte/macrophage precursors derived from bone marrow. CSF1 plays an important role in osteoclast formation with receptor activator of nuclear factor kappa-B ligand (RANKL) [15]. Because PVNS tissue expresses both CSF1 and RANKL [5], higher expression of these factors may result in the overexpression of osteoclast-like multinucleated cells and induce bone resorption and joint destruction in some cases. However, no studies have ever investigated the correlation of CSF1, CSF1R, and RANKL expression with the clinical features and course of PVNS.

In this study, we aimed to determine the expression of CSF1, CSF1R, and RANKL in PVNS tissues by immunohistochemistry and reveal the relationship between staining positivity and clinical features including occurrence of osteochondral lesions (bone erosion and/or osteoarthritis) and local recurrence after surgery. Additionally, we evaluated the number of giant cells and tumor volume and analyzed the association with various clinical variables.

## Materials and methods

### Clinical data

We collected clinical information from the patient database of 78 PVNS cases referred to our institutions between 1987 and 2014. Excluding cases with insufficient medical records, or not treated surgically, 40 cases, which were histologically diagnosed as PVNS by an experienced pathologist in our institution (YS) and had a specimen available for immunohistochemical analysis, were enrolled in this study. Information regarding age, sex, involved joints, occurrence of osteochondral lesions (bone erosion and osteoarthritis), and local recurrence were retrieved from the medical records. Institutional review board in our institution approved this study (approved number: 1332).

### Surgical treatment

Diffuse PVNS was treated as a rule with open tumor excision. Diffuse knee lesions were treated with open anterior and/or posterior total tumor excision until 2008. Subsequently, diffuse knee lesions were treated with arthroscopic anterior synovectomy and open excision of posterior lesions. In the localized type of PVNS, the tumor was simply resected en bloc. Patients received neither radiotherapy nor chemotherapy for PVNS.

### Immunohistochemistry

PVNS tissues were obtained at the time of surgery for immunohistological analysis. Tissues were fixed in 10 % formalin and embedded in paraffin. Immunohistochemical studies were conventionally performed using a streptavidin-biotin complex technique using formalin-fixed, paraffin-embedded sections. Paraffin specimens were cut at a thickness of 4 μm. The deparaffinized and rehydrated sections were treated with 0.3 % hydrogen peroxide in methanol for 15 min at room temperature to block the internal peroxidase activity followed by soaking in 10 % normal goat serum as a blocking agent for 10 min. The slides were incubated at 4 °C for 12 h with primary rabbit polyclonal antibodies, which have been confirmed to react with human target proteins. Primary antibodies were as follows: anti-CSF1 (ab9693; Abcam, Cambridge, UK; 1:100 dilution), anti-CSF1R (ab61137; Abcam, 1:100 dilution), and anti-RANKL (bs-0747R; Bioss MA, USA, 1:250 dilution). After rinsing with PBS, the sections were incubated with biotinylated anti-rabbit IgG conjugated with peroxidase as a second antibody, and the reaction products were observed using 3,3′-diaminobenzidine tetrahydrochloride. In cases in which a high deposition of hemosiderin was observed, Berlin blue staining was additionally performed. Slides were counterstained with hematoxylin, dehydrated, and mounted. Nonimmune goat serum was substituted for the primary antibody to serve as a negative control. Two orthopedic surgeons (TO, SH) and an experienced pathologist (YS) without knowledge of the clinicopathologic information evaluated the results of the immunohistochemical staining. Positive cells were counted individually in three different areas under a light microscope at ×400 magnification. Mean numbers of positively stained cells and total cells were calculated. The positive cell ratio (positive cell number/total cell number in the field) was determined. Positivity of the staining was divided into two groups: high and low according to the calculated mean value of the positive ratio.

### Evaluation of multinucleated giant cells

Since it is presumed that multinucleated giant cells have significant pathogenetic roles in not only the development of tumors but also of ostechondral lesions, their number was determined in 10 randomly selected fields in the specimen under a light microscope at ×100 magnification. We classified the results of the cell count as low (= < 50 cells/field) or high (>50).

### Assessment of tumor volume

We determined the tumor volume using magnetic resonance (MR) or computed tomography (CT) images in 29 measurable cases. Eleven cases were excluded, because of difficulty with the tumor volume measurement (not massive form) or a lack of imaging data. The maximum cross-sectional diameter (axial; R1, sagittal; R2, coronal; R3) was measured. We assumed the tumor to be oval-shaped and calculated the volume with a mathematical formula (4/3π × R1 × R2 × R3). In cases with multiple lesions, we added them up. The tumor volume was classified into two groups: small (= < 50 cm^3^) or large tumor (>50 cm^3^).

### Statistical analysis

Statistical analysis was performed using SPSS™ software, version 22.0. Fisher’s exact test and Pearson’s chi square test were applied to determine correlations between each of the variables (CSF1 expression, CSF1R expression, RANKL expression, the number of multinucleated giant cells, and tumor volume) and clinical features (gender, age, tumor localization, occurrence of osteochondral lesions, and recurrence of tumor). *P* values <0.05 were considered significant.

## Results

The mean age of patients was 35 years, ranging from 8 to 63 at the time of operation. Twenty-six patients were female and 14 were male. Knee joints were affected in 25 cases, hip in 6, foot and ankle in 4, elbow in 2, and shoulder, thigh, and sternocostal joints in one each (Table 1). Skeletal alterations (bone erosion and/or osteoarthritic change) were observed in 13 cases at the time of presentation or during the clinical course (6 cases: knee joints, 5; hip, 2; ankle 1). Three (2 hip joints, 1 knee joint) of 13 cases underwent total joint replacement surgery. Ten cases developed local recurrence (8 cases of knee joints, 2 of ankle joint).

**Table 1:** Demographic data of 40 cases

Variables	*N* (%)
Male	14 (35.0)
Female	26 (65.0)
Age, years (range)	35 (8–63)
Localization	
Shoulder	1 (2.5)
Elbow	2 (5.0)
Hip	6 (15.0)
Thigh	1 (2.5)
Knee	25 (62.5)
Ankle and foot	4 (10.0)
Sternocostal joint	1 (2.5)
Osteochondral change	
Yes	13 (32.5)
Hip^a^	5/6 (83.3)
Knee^a^	6/25 (24.0)
Ankle^a^	2/4 (50.0)
No	27 (67.5)
Local recurrence	
Yes	10 (25.0)
Knee^a^	8/25 (32.0)
Ankle^a^	2/4 (50.0)
No	30 (75.0)

^a^Each value is a ratio in each location

### Immunohistochemical staining for CSF1, CSF1R, and RANKL

#### CSF1 expression

**Fig. 1:**
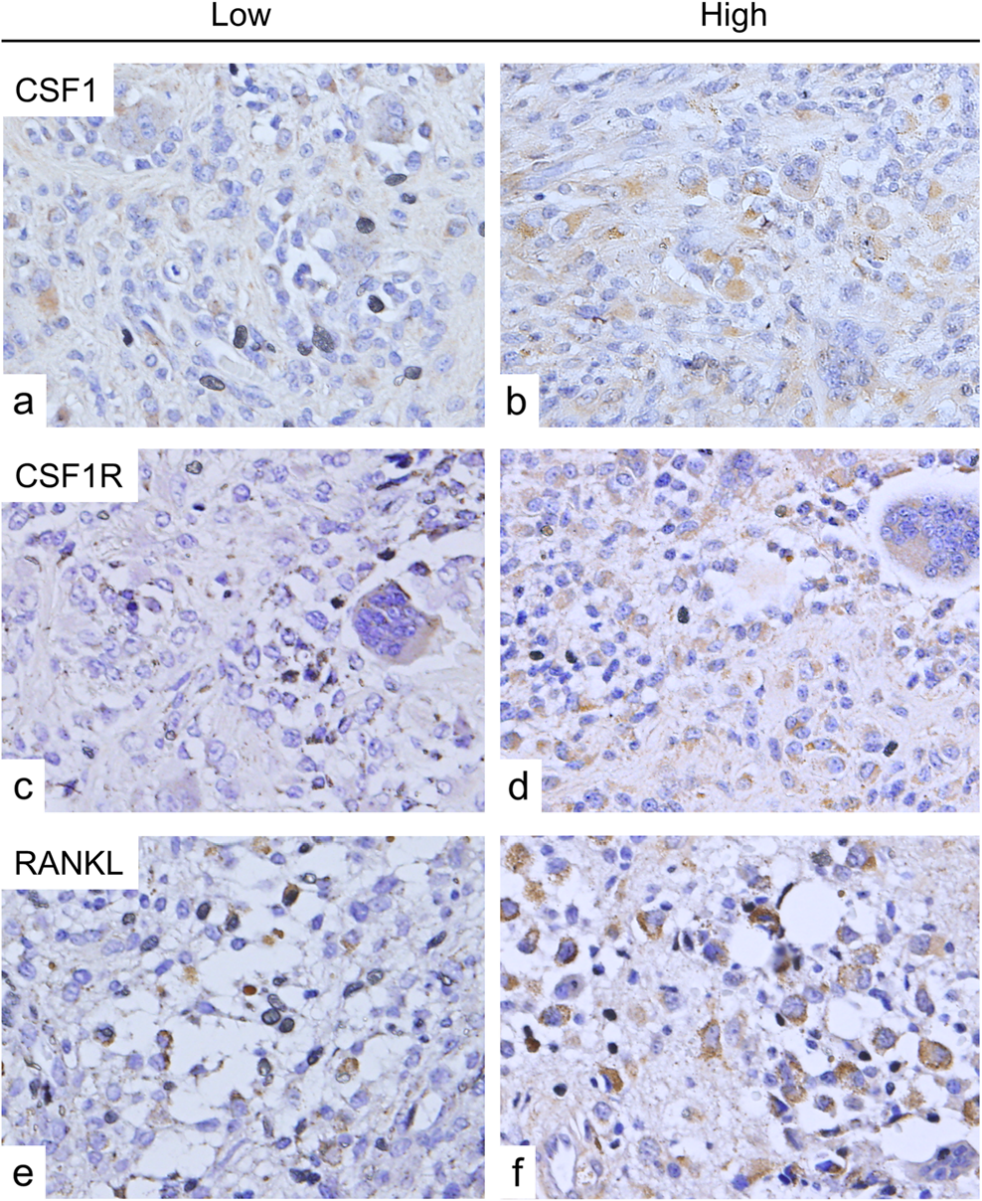
Immunohistochemical staining of PVNS. Photomicrographs show immunostaining of CSF1 (**a**, **b**), CSF1R (**c**, **d**), RANKL (**e**, **f**) in PVNS tissues. Low (**a**, **c**, **e**) and high (**b**, **d**, **f**) positivity of expression (counterstain with hematoxylin; original magnification, ×400)

CSF1 was positive in all cases (Fig. 1). Positive staining was observed mainly in mononuclear cells and some multinuclear giant cells. Mean positive rate was 7 % (range 3–15 %). Positivity of CSF1 staining was classified as high or low according to the cutoff value of 7 %. The cutoff value determined by receiver operatorating characteristic value analysis was also 7 %, suggesting that this value was adequate. In the 13 cases with osteochondral lesions, high positivity of CSF1 staining was observed in 11 cases (85 %). High positivity of CSF1 was significantly correlated with osteochondral lesions (*p* = 0.009) (Table 2), but not with local recurrence (*p* = 1) (Table 3). CSF1 immunostaining positivity was not statistically correlated with the number of giant cells, tumor volume, gender, age, or tumor localization.

**Table 2:** Factors correlated with the occurrence of osteochondral lesions

Variables	Osteochondral lesions		*p* value^a^
	No	Yes	
CSF1 expression			0.009
Low	16	2	
High	11	11	
CSF1R expression			1
Low	9	4	
High	18	9	
RANKL expression			0.298
Low	15	10	
High	12	3	
The number of giant cells			0.577
Low	12	7	
High	15	6	
Tumor volume			0.158
Small	17	5	
Large	3	4	

^a^Fisher’s exact test and Pearson chi square test were used to examine correlation with each variables

**Table 3:** Factors correlated with the recurrence of tumors

Variables	Local recurrencee		*p* value^a^
	No	Yes	
CSF1 expression			1
Low	14	4	
High	16	6	
CSF1R expression			0.124
Low	12	1	
High	18	9	
RANKL expression			1
Low	19	6	
High	11	4	
The number of giant cells			0.473
Low	13	6	
High	17	4	
Tumor volume			1
Small	16	6	
Large	5	2	

^a^Fisher’s exact test and Pearson chi square test were used to examine correlation with each variable

#### CSF1R expression

CSF1R was positive in all cases (Fig. 1). CSF1R stainability varied widely among cases and areas. Positive staining was observed in mononuclear and multinuclear giant cells (Fig. 1). The mean positive CSF1R staining rate was 10 % (range 6–22%). Positivity was divided into high and low groups according to the mean positive value (10 %). None of the clinical variables analyzed were significantly correlated with CSF1R positivity including osteochondral lesions (*p* = 1) (Table 2). CSF1R staining intensity was observed in 9 (90 %) of the 10 cases with local recurrence; however, it did not reach statistical significance (*p* = 0.124) (Table 3). Positivity of CSF1R immunostaining was not statistically correlated with the number of giant cells, tumor volume, gender, age, or tumor localization.

#### RANKL expression

Positivity for RANKL was observed in all cases. Some mononuclear cells and multinuclear giant cells were stained (Fig. 1). The mean value of the positivity was 9 % (range 4–15 %). Interestingly, RANKL positivity was not statistically correlated with the occurrence of osteochondral change including bone erosion (Table 2), recurrence of tumors (Table 3), the number of giant cells, tumor volume, gender, age, or tumor localization.

### Multinucleated giant cells

**Fig. 2:**
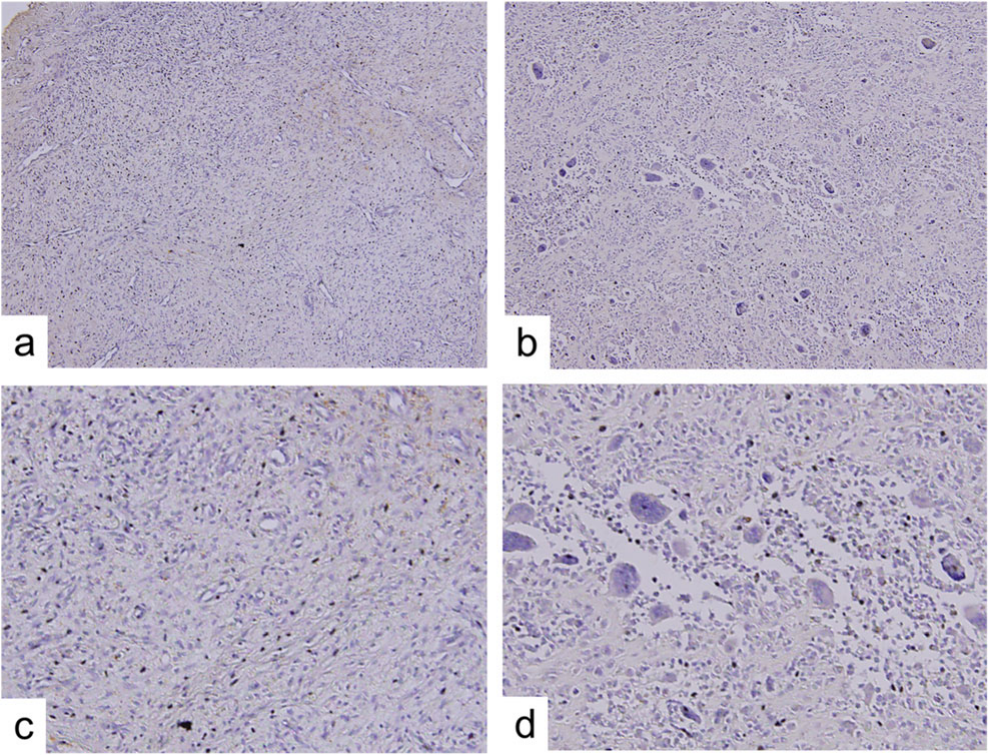
Multinucleated giant cells in PVNS. Photomicrographs show representative samples to count the number of giant cells. No multinucleated giant cells are observed, even at high magnification (**a**, **c**). A large number of multinucleated giant cells are seen, even at low magnification (**b**, **d**) (original magnification, ×100 (**a**, **b**), ×200 (**c**, **d**))

The number of multinucleated giant cells varied widely among cases. The mean number of multinucleated giant cells was 67/field ranging from 0 to 243 (Fig. 2). The number of multinucleated giant cells was divided into high and low groups according to the mean numbers. There was no significant difference in the number of giant cells between the presence and absence of osteochondral change (Table 2), recurrence of tumors (Table 3), gender, age, or tumor localization.

### Tumor volume

In 29 cases with measurable tumor volume, the median tumor volume was 48 cm^3^ ranging from 2 to 330 cm^3^. The tumor volume was divided into small and large groups according to the mean volume. Statistical analyses revealed that tumor volume was not associated with osteochondral change (Table 2), local recurrence (Table 3), gender, age, or tumor localization.

### Subgroup analysis

We focused on cases with knee joint involvement, because the highest rates of incidence and recurrence were observed in this joint in the present study. All of the six cases developing osteochondral lesions showed high positivity for CSF1 staining (*p* = 0.02) (Table 4). All of the eight cases with local recurrence showed high positivity for CSF1R staining, but without reaching statistical significance (*p* = 0.129) (Table 5). RANKL staining intensity was associated with neither increased occurrence of osteochondral lesions (*p* = 1) nor local recurrence (*p* = 0.667). The number of giant cells and tumor volume were not associated with osteochondral lesions or tumor recurrence.

**Table 4:** Factors correlated with the occurrence of osteochondral lesions in knee cases

Variables	Osteochondral lesions		*p* value^a^
	No	Yes	
CSF1 expression			0.02
Low	11	0	
High	8	6	
CSF1R expression			1
Low	5	1	
High	14	5	
RANKL expression			1
Low	11	4	
High	8	2	
The number of giant cells			0.645
Low	9	4	
High	10	2	
Tumor volume			1
Small	10	3	
Large	3	1	

^a^Fisher’s exact test and Pearson chi square test were used to examine correlation with each variables

**Table 5:** Factors correlated with the local recurrence of tumors in knee cases

Variables	Local recurrence		*p* value^a^
	No	Yes	
CSF1 expression			1
Low	8	3	
High	9	5	
CSF1R expression			0.129
Low	6	0	
High	11	8	
RANKL expression			0.667
Low	11	4	
High	6	4	
The number of giant cells			0.673
Low	8	5	
High	9	3	
Tumor volume			0.584
Small	9	4	
Large	2	2	

^a^Fisher’s exact test and Pearson chi square test were used to examine correlation with each variable

## Discussion

Problems developing in the clinical course of PVNS include ostechondral destruction [3] and high recurrence rate [16, 17]. Previous studies reported some risk factors possibly implicated in the aggressive behavior of PVNS (osteochondral destruction and high recurrence rate) including expression of matrix metalloproteinases (MMPs) [18, 19] and osteoclast-like cells induced by CSF1 and RANKL [14]. However, no previous studies have analyzed the relationship between the aggressive behavior of PVNS and expression patterns of cell types positive for CSF1, CSF1R, and RANKL, which might be pathogenetic determinants of PVNS [10, 12, 14]. We speculated that these molecules might be possible prognosticators of the aggressiveness of PVNS. The present study analyzed for the first time the possible correlation between the expression levels of CSF1, CSF1R, and RANKL with the clinical course of PVNS and demonstrated that high expression of CSF1 was significantly correlated with a high incidence of osteochondral change, while cases with high expression levels of CSF1R tended to have a high rate of local recurrence of knee PVNSs. Although other factors may be involved in the development of osteochondral destruction [3, 14, 18, 20], the findings of the present study may provide novel insights into the roles of CSF1 and CSF1R regarding not only the biological profiles of PVNS but also underlying data for assessing the efficacy of future biological agents for PVNS such as anti-CSF1R antibody.

The PVNS synovial tissue contains diverse cell types, composed of mononuclear cells and multinucleated giant cells. Several previous studies analyzed the cell characterization and cytokine expression in PVNS. Yoshida et al. investigated characteristics of cells constituting PVNS [5]. In their results, CSF1 and RANKL were expressed in both mononuclear and multinuclear giant cells, which were positive for CD68, a macrophage/histiocyte marker. Multinucleated giant cells expressed osteoclastic markers including TRAP, suggesting that mononuclear cells mediate the differentiation of osteoclasts from mononuclear cells via expression of CSF1 and RANKL. Several other studies also determined the characteristics of macrophage marker CD14 positive and negative mononuclear cells and multinucleated giant cells in PVNS [14, 21]. Taylor et al. showed that multinucleated giant cells are formed from CD14 positive cells and express an osteoclast phenotype. CD14 negative mononuclear cells that express RANKL and CSF1 support osteoclast formation [14]. The results of their study also indicated that osteoclast formation occurred even in the absence of exogenous CSF1, suggesting that overexpression of CSF1 in component cells might have a pathogenic role in PVNS. These findings are in part consistent with those of the present study in which osteochondral destruction occurred more often in cases with higher expression of CSF1.

In the experience of many physicians, osteochondral destruction does not develop in so many PVNS cases, while the frequency of osteochondral change is highly correlated with the site of PVNS involvement. A previous study identified a factor influencing osteochondral destruction in PVNS patients, namely, a limited joint space such as that present in the hips, feet, and ankles, which is significantly correlated with the occurrence of osteochondral destruction [3]. In the current study, most of the cases with hip joint involvement developed bone destruction and/or osteoarthritic change. We considered that a subgroup analysis should be performed focusing on cases with knee joint involvement, where the factor of joint volume might not affect the occurrence of osteochondral destruction. CSF1 was still significantly associated with the occurrence of osteochondral destruction in knee cases and was considered to be a risk factor. Interestingly, tumor volume was not associated with the occurrence of osteochondral events in knee joint, which has a large joint capacity (Table 2).

PVNS has a high local recurrence rate despite being a benign neoplasm [16, 22]. However, no investigations have yet analyzed the relationship between the molecular pathogenetic background, such as a CSF1/CSF1R signaling pathway, and the local recurrence rate of PVNS. Translocation of CSF1 in PVNS and subsequent CSF1 overexpression have been implicated in the pathogenesis of PVNS [9, 12]. West et al. described that CSF1-overexpressed tumor cells recruit CSF1R positive stromal cells through autocrine and paracrine pathways, leading to tumor formation, known as a “landscape effect” [10]. Large numbers of CSF1R positive cells in PVNS may reflect the higher recruitment activity of tumors. The results of the present study focusing on knee cases, where a higher relapse rate was observed, revealed that CSF1R expression was high in all relapsed knee cases, but not significantly so (*p* = 0. 129) probably due to the small numbers of cases. Further study is needed to obtain the definitive correlation between CSF1R expression and local recurrence.

Recently, several studies have investigated CSF1R as a possible therapeutic target in PVNS [23–27]. Blay et al. reported that treatment with imatinib mesylate achieved a complete response in cases of recurrent PVNS [26]. Cassier et al. reported the efficiency of imatinib mesylate in the treatment of locally advanced PVNS [27]. However, these recent studies did not investigate the predictive value of the expression level of CSF1 or CSF1R regarding the efficacy of the agent, while the present study provides novel information on the predictive value of pathogenetic molecules in the clinical course of PVNS.

The present study has several limitations. Due to the rarity of this disease, the study population was composed of only a small number of cases and a retrospective cohort. Larger number of cases is required to draw a definitive conclusion by multi-institutional study. There were several cases of diffuse type PVNS, which were excluded from the measurements of tumor volume because of the difficulty in evaluating this type. The results of the immunohistochemical analysis may have been influenced by the heterogeneity of the obtained samples, sensitivity of the antibody, and staining protocol. However, the immunohistochemistry results would be more consistent if performed with the same antibody, protocol, and skilled technique.

In conclusion, the present study, for the first time, investigated the possible correlation between the clinical course of PVNS and expression of pathogenetic molecules including CSF1 and CSF1R. The results suggested that a high positivity of CSF1 staining may predict the occurrence of osteochondral destruction in PVNS. Further investigation may help to clarify not only the possible correlation of CSF1 and CSF1R expression and clinical course of PVNS, but also the predictive value of the expression, thereby facilitating the development of optimal molecular target therapies.
